# Influence of Morphological Changes in a Source Material on the Growth Interface of 4H-SiC Single Crystals

**DOI:** 10.3390/ma12162591

**Published:** 2019-08-14

**Authors:** Matthias Arzig, Johannes Steiner, Michael Salamon, Norman Uhlmann, Peter J. Wellmann

**Affiliations:** 1 Crystal Growth Lab, Materials Department 6 (i-meet), Friedrich-Alexander-Universität FAU Erlangen-Nuremberg, D-91058 Erlangen, Germany; 2 Development Center for X-Ray Technology (EZRT), Fraunhofer Institute for Integrated Circuits, D-90768 Fürth, Germany

**Keywords:** in-situ CT, Laser flash, thermal conductivity, source material, numerical modelling, Computer Tomography, supersaturation, growth kinetics

## Abstract

In this study, the change of mass distribution in a source material is tracked using an in situ computer tomography (CT) setup during the bulk growth of 4H- silicon carbide (SiC) via physical vapor depostion (PVT). The changing properties of the source material due to recrystallization and densification are evaluated. Laser flash measurement showed that the thermal properties of different regions of the source material change significantly before and after the growth run. The Si-depleted area at the bottom of the crucible is thermally insulating, while the residual SiC source showed increased thermal conductivity compared to the initially charged powder. Ex situ CT measurements revealed a needle-like structure with elongated pores causing anisotropic behavior for the heat conductivity. Models to assess the thermal conductivity are applied in order to calculate the changes in the temperature field in the crucible and the changes in growth kinetics are discussed.

## 1. Introduction

The physical vapor deposition (PVT) growth technique has matured to being the standard process for the production of 4H silicon carbide (SiC) crystals [1]. With the aim of driving down the cost of SiC-based devices, great efforts have been made to increase the diameter size of SiC crystals. Wafer diameters of 150 mm are commercially available and the increase to diameters of 200 mm is in development [2]. Besides the required increase of boule size, the reduction of defects as well as bow and warp are important in order to compete with other materials [3]. A simple increase of the dimensions of the growth setup is not possible because increasing radial temperature differences have to be considered [4]. For the hot zone design, the reduction of thermal stress in the growing crystal while maintaining overall growth conditions with reasonable growth rates is crucial. In order to evaluate the growth conditions in the crucible, numerical simulation is the tool of choice [5,6,7,8]. One problem for the modeling of the growth conditions of bulk SiC is the lack of precise material data at elevated temperatures [9]. Another difficulty is accounting for the changes that happen inside the crucible during the growth process. One approach is to set up a model in order to find how the source material evolves during growth and compare it with the experimental results afterwards [6].With our in situ Computer Tomography (CT) system [10], we are able to track the changes in the experiment, despite the very high process temperatures. From several growth runs in our laboratory we know that the powder source changes significantly during the experiment. Our new approach is to take the changes in the mass distribution from the in situ CT measurements as a basis for our numerical model. With this work we want to examine how the properties of the source material change and to which extend these changes will affect the growth conditions.

## 2. Materials and Methods

For the study, 3-inch 4H-SiC crystals were grown in our standard PVT growth setup. A graphite crucible was heated inductively and a thermal gradient between top and bottom was employed in order to facilitate mass transport from the source area at the bottom to the seed mounted at the top. A temperature of 2120 °C was measured on top of the crucible. In order to control the growth rate, an Argon background pressure of 10 mbar was established. For the n-type doping of the growing crystal, 10% Nitrogen was added to the gas flow. The total flow rate of the gas was set to 110 sccm. The crystal grew 16 mm in length with a growth rate of around 130 µm/h.

The progress of the crystal growth experiment is imaged in intervals using our in situ CT system. This consists of a 125 kVp DC tungsten anode tube (SB-125-700-P, Source-Ray, Inc) radiating through the growth setup onto a digital flat panel X-Ray detector (PaxScan 2520 D/CL, Varian Medical Systems). By rotation of the inner setup, X-ray images can be taken from every angle, enabling calculation of the 3-dimensional mass distribution in the crucible [10]. Therefore, 400–800 2D projections captured during 360° rotation were processed by the Feldkamp-Algorithm. As it takes only 40 min to perform a single CT measurement, the growth interface can be depicted accurately due to the small growth rates.

In order to measure the change of thermal properties, specimens were taken from different locations in the used source material. Two samples taken from the bottom and two samples from the central region of the used feed stock were prepared. To determine the thermal properties before growth, a different sample of the used SiC source powder was heated to growth temperature and cooled down without performing crystal growth. All samples were shaped into small blocks of 10 mm × 10 mm × 5–6 mm dimensions. The thermal diffusivity was measured using a LINSEIS LFA 1000 *l*aser flash analyzer (Linseis Messgeraete GmbH, 95100 Selb, Germany). A good description of the laser flash method can be found in previous work [11]. Five measurements of each sample were conducted at room temperature and at elevated temperatures between 100 °C and 1200 °C at intervals of 100 °C in a vacuum atmosphere.

For the accurate determination of the structure of the two samples from the center of the feed stock, ex situ CT scans were conducted using a high resolution CT setup. This consisted of a FXE 225.99 Twin Head X-Ray source (150 kV, 150 mA) and a Thales medical flat panel detector. In total, 1600 projections were captured and processed with the Feldkamp-Algorithm, yielding a 3D dataset of the structure with a voxel size of 6.52 µm.

## 3. Results

As was shown before with the in situ CT [12], the source material in the crucible undergoes alterations with ongoing growth time. Starting from the homogeneous distributed powder of more or less spherical particles, the temperature gradient in the crucible induces a morphology change. Sublimation and recrystallization lead to the formation of elongated needle-like structures aligned in the direction of heat flow. The source material is consumed first in the hottest areas at the bottom of the crucible. Due to the difference in partial pressures, the reactive species (Si, SiC_2_, Si_2_C, etc.) sublime incongruently [13,14,15]. Therefore, in the hottest areas a graphite skeleton without Si is left behind.

### 3.1. Thermal Conductivity

**Figure 1:**
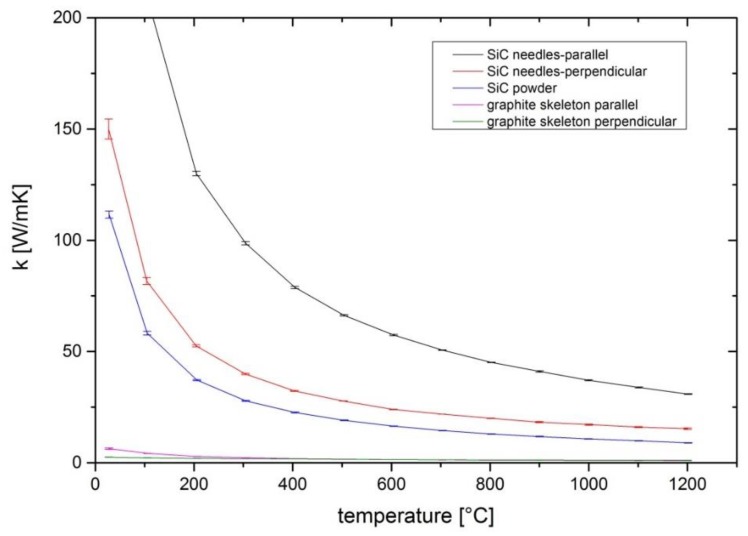
Thermal conductivities at different temperatures measured by laser flash. The connecting lines between the measurement points serve as a guide for the eye.

The change in density, morphology, and also composition leads to a difference in thermal conductivity. Figure 1 shows that the samples taken from the used source differ strongly from the initial conductivity of the powder filled into the crucible. With the laser flash method the thermal diffusivity α can be obtained, and together with the density ρ and the specific heat capacity c_p_ the thermal conductivity k can be calculated by:

k = α × ρ × c_p_, (1)

For the calculation of the thermal conductivities of the SiC powder and the needle-like structured SiC samples, the specific heat capacity of single crystalline SiC was taken from previous work [16], and for the Si-depleted graphite skeleton the specific heat capacity of graphite taken from another study [17] was used. The densities of the samples are listed in Table 1. The different densities illustrate the change of mass distribution in the source area during growth. A densification takes place in the top part, while the graphite skeleton at the bottom exhibits an eminently low density. The thermal conductivity of ~1 W/mK at 1200 °C for both graphite skeleton samples demonstrates that the thermal conductivity is diminished severely in the bottom area compared with the initial value of ~9 W/mK at 1200 °C for the SiC powder. 

**Figure 2:**
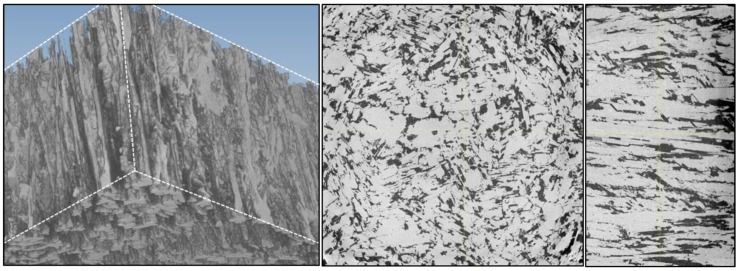
High resolution 3D CT data of the sample taken from the top of the source material with the SiC needles parallel to the heat flow in the laser flash measurement. On the left a 3D view is depicted. In this representation the SiC fraction is transparent in order to illustrate the alignment of the elongated pores depicted in grey. In the middle a 2D cut horizontally through the CT data is depicted, showing the SiC proportion in black and the pore proportion in grey. The right side shows a longitudinal view of the SiC needles arranged in parallel to each other. The SiC material is grey, while the pores are depicted in black.

With an increasing density in the top part of the source, the thermal conductivity also increases. Additionally, a strong dependence of the sample orientation in the measurement setup becomes apparent. In order to illustrate the reason for this behavior, ex situ CT measurements were conducted. In Figure 2, the 3D data is depicted showing the pore structure after growth. The pores are more or less cylindrical in their shape and they are well-aligned along the direction of heat flow in the crucible. The shape and alignment of the pores is the reason for the difference in thermal conductivity depending on the measurement direction. The sample with the highest conductivity in Figure 1 was prepared in a way that the pores are aligned parallel to the direction of heat flow for the laser flash measurements. This means the SiC needles are well-aligned in the direction of heat flow, enabling the heat conduction. The sample denoted as “SiC-needles-perpendicular” was cut in a way that all the cylindrical pores are aligned perpendicular to the direction of heat flow in the laser flash measurement. In that case the heat flow through the solid is strongly impaired by the pores. 

### 3.2. Models for the Thermal Conductivity

In order to describe the properties of the source material, the thermal conductivity of the SiC needles as well as that of the pores have to be taken into account. In a previous study [18], Loeb presents an equation to describe the effective thermal conductivity of a pore dependent on its geometry:

(2) $$k_{\mathrm{pore}}=4{\gamma d\varepsilon \sigma T}^{3}$$

The γ denotes a geometrical factor accounting for the geometry of the pore. For laminar and cylindrical pores with the axis in parallel to the heat flow it becomes γ = 1. If the heat flow is perpendicular to the cylindrical pores, γ = π/4 has to be used. For the case of spherical pores, γ = 2/3 can be used. Besides the shape, the largest dimension of the gap in the direction of the heat flow is taken into account with d. As the heat transfer in the pore is radiative, the emissivity ε of the material, the Stefan-Boltzmann constant σ, as well as the Temperature T are included. 

**Figure 3:**
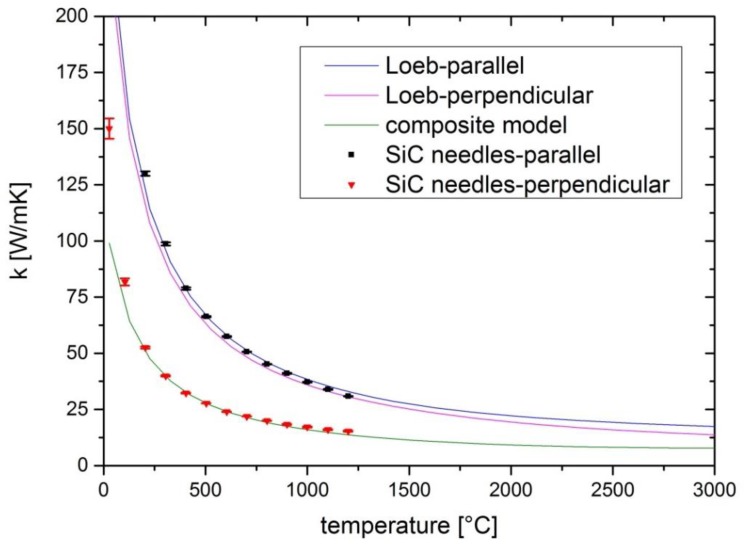
Measured thermal conductivities of the samples taken from the used source material. The red triangles denote the sample prepared with the SiC needles perpendicular to the heat flow and the black squares indicate the sample prepared with the SiC needles parallel to the heat flow in the laser flash experiment. The blue and purple lines denote the application of the Loeb model [18] in the parallel case and in the perpendicular case, respectively. The green line represents the composite model taken from a previous study [19].

For the effective thermal conductivity k_p_ of a material containing pores, Loeb developed the following Equation:

(3) $$k_{p}=k\left(1-P_{c}\right)+\frac{P_{c}}{\frac{1}{k}\left(1-P_{L}\right)+\frac{P_{L}}{4{\gamma \varepsilon \sigma \mathrm{dT}}^{3}}}$$

where k is the thermal conductivity of the solid, P_c_ is the pore fraction in the plane perpendicular to the heat flow, and P_L_ is the longitudinal pore fraction. For the thermal conductivity of the solid we take the values of SiC from a previous study [16]. With the porosities calculated from the ex situ CT measurements, the model can be applied for the samples taken from the used powder stock. As depicted in Figure 3, this model can describe the heat flow for the case of the SiC needles (and therefore also the pores) in parallel to the flow direction very accurately, but it overestimates the thermal conductivity for the perpendicular case. For the model a rigid material is assumed that only contains a few cylindrical pores. This assumed material still offers a large proportion of solid material without thermal conduction being hampered by pores. In our case, we have more isolated elongated SiC needles arranged next to each other with only a few touching points, as depicted in Figure 2. High resolution 3D CT data of the sample taken from the top of the source material with the SiC needles parallel to the heat flow in the laser flash measurement. On the left a 3D view is depicted. In this representation the SiC fraction is transparent in order to illustrate the alignment of the elongated pores depicted in grey. In the middle a 2D cut horizontally through the CT data is depicted, showing the SiC proportion in black and the pore proportion in grey. The right side shows a longitudinal view of the SiC needles arranged in parallel to each other. The SiC material is grey, while the pores are depicted in black. Therefore, the proportion of unperturbed thermal conduction through the solid material is much smaller for the case of heat flow perpendicular to the SiC needles. 

The structure of the source material can also be regarded as a composite of pores and solid SiC. In a previous study [19], Kulkarni and Brady described the thermal conductivity of composites in analogy to an electrical resistor model dependent on the orientation of fibers in a matrix. To utilize this analogy, in the case of heat flow perpendicular to the needle orientation, the source material can be regarded as a series connection of thermal resistances of the SiC needles and the pores. The thermal resistance is inversely proportional to the thermal conductivity, and therefore the thermal conductivity k_s_ for the composite is calculated by:

(4) $$k_{s}={\;k}_{m}\left[\frac{k_{f}\left(1+V_{f}\right)+{\;k}_{m}\left(1-V_{f}\right)}{k_{f}\left(1-V_{f}\right)-{\;k}_{m}\left(1+V_{f}\right)}\right]$$

While k_m_ and k_f_ denote the thermal conductivities of the matrix and the fiber, respectivley, V_f_ denotes the volume fractions of the fiber, which are the SiC needles in this case. As visible in the ex situ CT measurement, the SiC needles are not completely isolated from each other, but some connections between them have to be considered. These points of connection can serve for heat conduction and need to be included in the model with an additional connection in parallel:

(5) $$k_{p}={\;k}_{f}V_{f}+k_{m}\left(1-V_{f}\right)$$

With this equation we can take into account the fraction of heat flow in between the needles. For our sample, this fraction is estimated to be 0.3 in order to fit the measured values. As a result of combining the connection in series and in parallel, the model seems to be in good accordance to our measured values from the laser flash experiments, as denoted in Figure 3.

Because of the more complex structure of a packed bed and the geometry of the contacting spots, for the SiC powder it is challenging to identify a proper model. For an overview covering many models of heat conduction in porous medial, this can be found in previous work [20], and a very elaborate model for SiC powder can be found in another study [21]. In this work, a semi-quantitative approach was chosen. In principle the conductivity should be composed of a series connection of the radiative heat transfer between the particles and the heat conductivity from the bottom of the particle to its top. However, as the sintering between the particles already starts as soon as the growth temperature is reached, there should be some pathways for thermal conduction from particle to particle. As the particles are connected randomly to their neighbors, a 3-dimensional network of thermal conduction is formed. Therefore, again the model of the electrical resistor in the series will be used for the combined heat transfer of the pore fraction and the SiC particle fraction. On top, we include a proportion of thermal conductance of SiC to account for the 3-dimensional network of the particles weighted in order to fit to our measured values from the laser flash experiments.

### 3.3. Modeling of the Temperature Field 

For the modeling of the temperature field we use the commercial software COMSOL Multiphysics Version 5.2.a (COMSOL AB, Stockholm, Sweden), utilizing a Finite Element Method (FEM). The geometry is copied from our in situ CT measurements to rebuild the rotationally symmetric layout of the growth reactor with the current mass distribution inside the crucible. In order to evaluate the influence of the anisotropic source material, a 2 × 2 tensor is used to describe the directional thermal conductivity k. The tensor consists of the temperature dependent thermal conductivities described in the previous section, distributed on the diagonal of the 2 × 2 array. 

**Figure 4:**
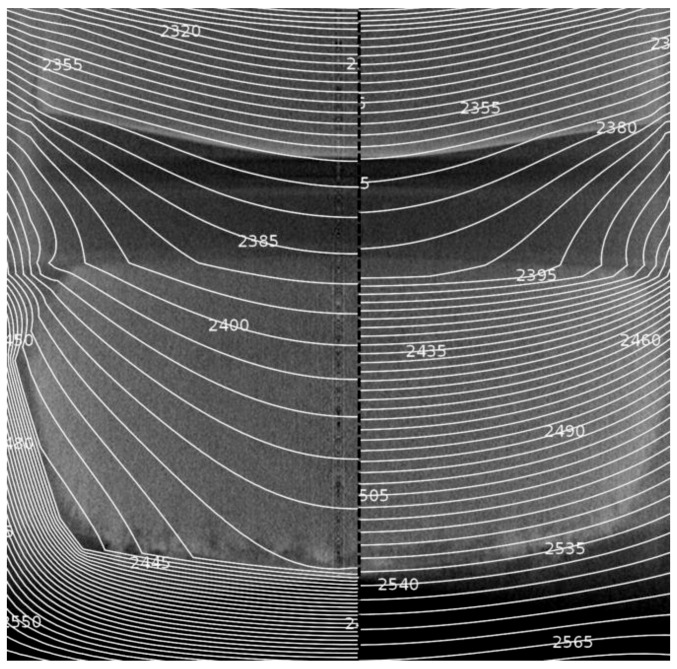
In situ CT data for the crucible at a growth time of 115 h with an overlay indicating the results from the numerical modeling of the temperature field. On the left half, the discussed thermal properties of the graphite skeleton and the anisotropic needle-like structure are taken into account for the source area. In comparison, the right side shows the temperature field if the initial thermal conductivity of the SiC powder is assumed in the whole source area.

In Figure 4 the influence of the changing thermal properties on the temperature distribution in the crucible is depicted. The left side depicts the modeling with the applied findings discussed before, while the right side should illustrate the difference if we ignore the changes in the source. Apparently, the Si-depleted area at the bottom of the powder source has a big influence on the temperature distribution. It almost acts as insulation and shields the remaining SiC source material from the hot crucible walls and the bottom. Therefore, the maximum temperature of the remaining SiC source is strongly reduced as compared to the temperatures at the bottom of the source area in the beginning of the crystal growth experiment. As the thermal conductivity of the remaining source material is increased, especially in the vertical direction, the heat is conducted more easily, leading to smaller thermal gradients in the source material. If the changes of the source material during growth are not taken into account for the simulation, the maximum temperatures in the source material are overestimated, as depicted in the right side of Figure 4.

**Figure 5:**
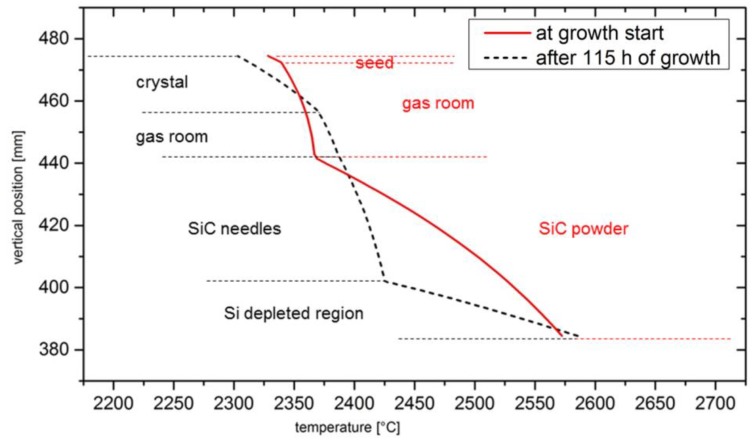
Comparison of the temperatures inside the crucible in the beginning (red line) of the experiment with the temperatures after 115 h of growth (dotted black line). The temperatures were taken in the vertical direction in the rotational center of the crucible from the bottom of the powder to the backside of the crystal. The horizontal lines indicate the boundaries between different types of materials in the crucible.

These findings indicate that the morphological changes strongly affect the growth conditions. In order to estimate the differences, a simulation of the temperature field of the growth start consisting of the isotropic powder in the bottom and the thin seed at the top is performed. A line profile of the temperature from the bottom of the powder to the backside of the seed was taken to demonstrate the vertical temperature distribution, as depicted in Figure 5. 

At the bottom of the source area the temperatures only differ by approximately 10 °C for the two cases. As the very small slope of the black dotted line indicates, the Si-depleted region insulates the remaining powder charge from the hot crucible bottom. Therefore, the maximum temperature is much higher in the source material at the growth start. The temperature at the bottom of the powder is estimated to be 2570 °C at the growth start, while the bottom of the remaining source material has a temperature of 2425 °C after 115 h of growth. However, the increased heat conductivity due to the morphological changes in the SiC source leads to a higher temperature of 2390 °C at the top of the SiC charge compared to 2370 °C in the beginning of the experiment. Also, the temperatures at the growth front differ significantly for the two cases. As the crystal grows into the gas room its surface temperature increases from initially 2340 °C to 2370 °C after 115 h. 

From these variations of the temperatures during the growth we can conclude that the growth kinetics change significantly during the experiment. The growth of the crystal is enabled by the supersaturation of the gaseous species at the growth front. The supersaturation depends on the transport of the vapor species to the growth front and on the local partial pressure of the species. The transport mainly depends on the background pressure, as we are growing in the diffusion limited regime. In our experiment, the Ar pressure is kept constant at 10 mbar throughout the whole growth time. However, the partial pressures of the reactive species depend strongly on the local temperatures inside the growth chamber. We consider the species SiC_2_ to be growth limiting in our system and calculate its temperature depending partial pressure from previous work [15]. In order to illustrate the changes in growth kinetics, we will use a simplified measure of the supersaturation

(6) $$S=\frac{p_{s}-p_{c}\;}{{\;p}_{c}}$$

where p_s_ denotes the partial pressure at the top of the source and p_c_ the partial pressure at the growth interface. It turns out that the supersaturation decreases with increasing growth time from 52% at the start to 31.5% after 115 h because the temperature difference between the source and crystal decreases. This would mean that the growth velocity of the crystal slows down, but we do not see obvious evidence of that in our experiment. With our simplification we neglected the influence of the transport process in the gas phase. In the experiment the distance between source and seed becomes smaller as the crystal grows, and therefore the diffusion of the gaseous species through the gas room is faster, leading to a more constant growth rate than expected from the change in supersaturation. Determined from the vapor pressures of Si, Si_2_C, and SiC_2_ at the top of the powder, the C/Si ratio increases from 0.33 to 0.35, indicating a slight increase in Si content.

With these considerations the overall growth conditions seem to be relatively constant, despite the strong changes visible in the source material area. Interestingly, at the growth start, the vapor pressure of SiC_2_ at the bottom of the source is calculated to be 22.2 mbar and the vapor pressures of all gaseous SiC species add up to 88.5 mbar. When the Ar pressure is reduced from 100 to 10 mbar in order to start the growth, this pressure could lead to a boost of reactive species pushed through the powder into the gas room, leading to a short period of raised supersaturation. This effect should be very short, as the recrystallization in the source area forms a balance of evaporation and recrystallization. An indication for this may be found in a previous study [22], where a very homogeneous growth rate throughout the whole PVT growth of SiC is reported, but in the first hour of growth a strongly increased growth rate is visible. As soon as the dense disk on top of the powder is formed, the growth rate drops and stays rather constant. 

A strong influence of the source material on the shape of the crystal can be found if the growth is extended and the remaining powder source shrinks laterally, as we reported in previous work [12]. A larger horizontal temperature gradient in the source material as well as more localized evaporation of the source material in the center lead to a significantly bent surface. Therefore, a powder with high packing density and rather large grains seems to be favorable in order to maintain stable growth conditions, as the changes inside the source material will elapse in a more constant manner.

## 4. Conclusions

We showed that the source material transforms significantly during the growth process, and therefore its density and thermal properties change alike. The needle-like structure that forms in the source area has an increased thermal conductivity compared to the powder initially loaded into the growth chamber. Because of its microstructure, the altered source material exhibits an anisotropic thermal conductivity. The different thermal conductivities of the changing source material were determined with laser flash measurements. Several models that describe the thermal conductivities are compared and taken into account for the numerical modeling of the temperature field. Compared to previous reports, the presented work considers the anisotropic changes of the SiC source material quantitatively. 

It turned out that the overall growth conditions do not change severely if the transformations in the source material are considered. If the changes in the source area are neglected, the temperature on top of the powder, and therefore also the supersaturation, would be overestimated. We found an indication that a large pressure gradient in the powder could influence the initial growth phase just after the start of the experiment. 

## Figures and Tables

**Table 1:** Densities (in kg/m^3^) used for the calculation of the thermal conductivities. The density is calculated by dividing the whole volume of the sample (pore volume is not subtracted) by its weight.

	SiC Needles Parallel	SiC Needles Perpendicular	SiC Powder	Graphite Skeleton Parallel	Graphite Skeleton Perpendicular
density (kg/m^3^)	2390	2290	1320	110	142
Deviation (kg/m^3^)	±4.90	±2.11	±8.82	±1.32	±1.37
